# Selective targeting of angiopoietin-like 3 (ANGPTL3) with vupanorsen for the treatment of patients with familial partial lipodystrophy (FPLD): results of a proof-of-concept study

**DOI:** 10.1186/s12944-021-01589-4

**Published:** 2021-12-05

**Authors:** Maria C. Foss-Freitas, Baris Akinci, Adam Neidert, Victoria J. Bartlett, Eunju Hurh, Ewa Karwatowska-Prokopczuk, Elif A. Oral

**Affiliations:** 1 Division of Metabolism, Endocrinology & Diabetes and Caswell Diabetes Institute, University of Michigan, MI Ann Arbor, USA; 2 Dokuz Eylul University, İzmir, Turkey; 3 Akcea Therapeutics, Inc, MA Boston, USA; 4 Metabolism, Endocrinology and Diabetes, Department of Internal Medicine, Michigan Medicine, University of Michigan, Caswell Diabetes Institute, 2800 Plymouth Road, North Campus Research Complex, 25-3696, MI 48109-2800 Ann Arbor, USA

**Keywords:** Familial partial lipodystrophy, Angiopoietin-like protein 3, Triglycerides, Mixed meal test, Adipose tissue insulin resistance, Vupanorsen

## Abstract

**Background:**

Familial partial lipodystrophy (FPLD) is a rare disease characterized by selective loss of peripheral subcutaneous fat, associated with dyslipidemia and diabetes mellitus. Reductions in circulating levels of ANGPTL3 are associated with lower triglyceride and other atherogenic lipids, making it an attractive target for treatment of FPLD patients. This proof-of-concept study was conducted to assess the efficacy and safety of targeting *ANGPTL3* with vupanorsen in patients with FPLD.

**Methods:**

This was an open-label study. Four patients with FPLD (two with pathogenic variants in *LMNA* gene, and two with no causative genetic variant), diabetes (HbA1c ≥ 7.0 % and ≤ 12 %), hypertriglyceridemia (≥ 500 mg/dL), and hepatic steatosis (hepatic fat fraction, HFF ≥ 6.4 %) were included. Patients received vupanorsen subcutaneously at a dose of 20 mg weekly for 26 weeks. The primary endpoint was the percent change from baseline in fasting triglycerides at Week 27. Other endpoints analyzed at the same time point included changes in ANGPTL3, fasting lipids and lipoproteins, insulin secretion/sensitivity, postprandial lipids, and glycemic changes in response to a mixed meal test, HFF measured by MRI, and body composition measured by dual-energy absorptiometry (DEXA).

**Results:**

Baseline mean ± SD fasting triglyceride level was 9.24 ± 4.9 mmol/L (817.8 ± 431.9 mg/dL). Treatment resulted in reduction in fasting levels of triglycerides by 59.9 %, ANGPTL3 by 54.7 %, and in several other lipoproteins/lipids, including very low-density lipoprotein cholesterol by 53.5 %, non-high-density lipoprotein cholesterol by 20.9 %, and free fatty acids (FFA) by 41.7 %. The area under the curve for postprandial triglycerides, FFA, and glucose was reduced by 60 %, 32 %, and 14 %, respectively. Treatment with vupanorsen also resulted in 55 % reduction in adipose tissue insulin resistance index, while other insulin sensitivity indices and HbA1c levels were not changed. Additional investigations into HFF and DEXA parameters suggested dynamic changes in fat partitioning during treatment. Adverse events observed were related to common serious complications associated with diabetes and FPLD. Vupanorsen was well tolerated, and there was no effect on platelet count.

**Conclusions:**

Although limited, these results suggest that targeting ANGPTL3 with vupanorsen could address several metabolic abnormalities in patients with FPLD.

**Supplementary Information:**

The online version contains supplementary material available at 10.1186/s12944-021-01589-4.

## Background

Familial partial lipodystrophy (FPLD) is a rare disease characterized by selective loss of peripheral subcutaneous adipose tissue and redistribution, usually affecting the trunk and limbs, but preservation in other areas such as the face and neck [1]. It is usually associated with metabolic complications, including severe hypertriglyceridemia, hepatic steatosis, insulin resistance, diabetes mellitus and relatively low leptin levels [2]. The disease is heterogeneous in both presentation and etiology. Patients present with a wide array of multi-system abnormalities and carry a high disease burden that may lead to increased early mortality, predominantly due to cardiovascular causes [3]. There are numerous genes that cause the phenotype, as well as polygenic forms [1, 2, 4–6]. The diagnosis of FPLD can be established by careful clinical assessment of fat distribution through visual and physical examination [7], use of whole body dual-energy absorptiometry (DEXA) to accurately visualize and document the fat tissue distribution pattern [8], and genetic testing, when available [9]. FPLD can be viewed as an extreme form of metabolic syndrome and Type 2 diabetes (T2DM) associated with truncal obesity, but the residual fat is likely highly dysfunctional with more abnormalities due to the inherent genetic defects. Currently, there are no approved specific therapies for this disease in the US [4, 6].

Angiopoietin-like protein 3 (ANGPTL3) is a glycoprotein secreted by the liver that inhibits lipoprotein lipase and endothelial lipase activity, two key enzymes involved in the metabolism of triglyceride-rich lipoproteins (TRLs) and high-density lipoprotein (HDL), respectively [10–12]. Individuals with homozygous loss-of-function mutations in the *ANGPTL3* gene present with a phenotype of familial combined hypolipidemia Type 2 (FHBL2), characterized by decreased plasma levels of triglycerides, low-density lipoprotein cholesterol (LDL-C), and high-density lipoprotein cholesterol (HDL-C) [12–15]. In addition, they have a reduced risk of coronary artery disease (CAD) [13–17]. Other beneficial metabolic effects that have been observed include increased insulin sensitivity and reduction in circulating FFA levels [18]. A similar hypolipidemic profile has been achieved by treatment with antisense oligonucleotides (ASO) targeting *ANGPTL3* mRNA in the liver [19] or a monoclonal antibody against ANGPTL3 [14]. Therefore, we hypothesized that reduction of ANGPTL3 levels had the potential to reduce hypertriglyceridemia and associated metabolic abnormalities in FPLD patients.

Vupanorsen is a second-generation N-acetyl galactosamine (GalNAc3)-conjugated ASO, targeting hepatic *ANGPTL3* mRNA. The high affinity of vupanorsen for the hepatocyte-specific asialoglycoprotein receptor, confers therapeutic efficacy similar to that of the parent unconjugated compound but with 20- fold lower dosing, thus reducing systemic exposure [19].

Given the lack of therapeutic options for patients with FPLD, we sought to determine whether vupanorsen has the potential to address the multiple metabolic abnormalities in this disease. This proof-of-concept study in FPLD patients with hypertriglyceridemia, diabetes mellitus and hepatic steatosis, was designed to evaluate the effect of vupanorsen on lipid metabolism, glucose control, insulin resistance, and body fat distribution, including HFF. The study also assessed safety and tolerability of vupanorsen in FPLD patients.

## Methods

### Study design and patient population

This proof-of-concept, phase 2, open-label study enrolled 4 patients, aged 18 years or above with clinical diagnosis of FPLD, elevated fasting plasma triglycerides (≥ 500 mg/dL or ≥ 5.7 mmol/L), diabetes mellitus associated with lipodystrophy [Hemoglobin (Hb) A1c ≥ 7 % and ≤ 12 %], and hepatic steatosis with mean hepatic fat fraction (HFF) ≥ 6.4 % by magnetic resonance imagining (MRI). Diagnosis of lipodystrophy was based on a deficiency of subcutaneous body fat in a partial fashion, assessed by physical examination and low skinfold thickness in the anterior thigh by caliper measurement: men (≤ 10 mm) and women (≤ 22 mm). Patients were also required to have at least one of the following: genetic diagnosis of FPLD, family history of FPLD or family history of abnormal and similar fat distribution, plus one minor criterion or (in the absence of FPLD-associated genetic variant or family history) two minor criteria, and body mass index (BMI) < 35 kg/m^2^. Patients treated with anti-diabetic (except for GLP-1 agonists), lipid lowering, or atypical antipsychotic medications had to be on stable therapy for at least 3 months prior to screening. Detailed inclusion and exclusion criteria are listed in Table 1.

**Table 1:** Inclusion and exclusion criteria

Inclusion criteria	Exclusion criteria
Age ≥ 18 years at the time of informed consent	Diagnosis of generalized lipodystrophy or acquired partial lipodystrophy (APL)
Clinical diagnosis of familial partial lipodystrophy plus diagnosis of type 2 diabetes mellitus and hypertriglyceridemia. Diagnosis of lipodystrophy is based on deficiency of subcutaneous body fat in a partial fashion assessed by physical examination and low skinfold thickness in the anterior thigh by caliper measurement: men (≤ 10 mm) and women (≤ 22 mm), and at least 1 of the following: • Genetic diagnosis of familial PL (e.g., mutations in LMNA, PPAR-γ, AKT2, CIDEC, PLIN1 genes) OR • Family history of FPLD or family history of abnormal and similar fat distribution plus 1 Minor Criteria OR 2 Minor Criteria (In the absence of FPLD-associated genetic variant or family history) and BMI< 35 kg/m2 MINOR Criteria a. Requirement for high doses of insulin, e.g., requiring ≥ 200 U/day, ≥ 2 U/kg/day, or currently taking U-500 insulin b. Presence of acanthosis nigricans on physical examination c. Evidence/history of polycystic ovary syndrome (PCOS) or PCOS-like symptoms (hirsutism, oligomenorrhea, and/or polycystic ovaries) d. History of pancreatitis associated with hypertriglyceridemia e. Evidence of non-alcoholic fatty liver disease • Hepatomegaly and/or elevated transaminases in the absence of a known cause of liver disease or radiographic evidence of hepatic steatosis (e.g., on ultrasound or CT)	Medical history of: • Recently diagnosed with acute pancreatitis (4 weeks from Screening) • acute or unstable cardiac ischemia (myocardial infarction, acute coronary syndrome, new-onset angina), stroke, transient ischemic attack (6 months from screening) • major surgery within 3 months of Screening • History of heart failure with New York Heart Association functional classification (NYHA) greater than Class II • Uncontrolled hypertension (blood pressure [BP] > 160 mm Hg systolic and/or 100 mm Hg diastolic) • History of bleeding diathesis or coagulopathy or clinically significant abnormality in coagulation parameters at Screening • Active infection requiring systemic antiviral or antimicrobial therapy that will not be completed before Study Day 1 • Known history of or positive test for human immunodeficiency virus (HIV), hepatitis C or chronic hepatitis B • Malignancy within 5 years, except for basal or squamous cell carcinoma of the skin or carcinoma *in situ* of the cervix that has been successfully treated
Presence of diabetes mellitus associated with lipodystrophy [Hemoglobin (Hb) A1c ≥ 7 % and ≤ 12 %]	Clinically significant abnormalities in screening laboratory values that would render a subject unsuitable for inclusion, including the following: • Urine protein/creatinine ratio (UPCR) ≥ 0.25 mg/mg. • Estimated GFR ˂ 60 mL/min/1.73 m2 • Alanine aminotransferase (ALT) or aspartate aminotransferase (AST) > 2x ULN • Bilirubin > ULN • Alkaline phosphatase (ALP) > 1.5 X ULN • Platelet count ˂ LLN
Elevated fasting plasma triglycerides (≥ 500 mg/dL or ≥ 5.7 mmol/L)	Use of metreleptin or anti-obesity drugs within 3 months before screening, GLP-1 agonists or systemic corticosteroids or anabolic steroids within 4 weeks before screening
Presence of hepatosteatosis (fatty liver), as evidenced by a Screening MRI indicating a hepatic fat fraction (HFF) ≥ 6.4 %	History within 6 months of Screening of drug or alcohol abuse
Willing to maintain their customary physical activity level and to follow a diet moderate in carbohydrates and fats with a focus on complex carbohydrates and to replace saturated for unsaturated fats	Unwilling to comply with lifestyle requirements
Provided written informed consent	

*GFR* Glomerular filtration rate, *ULN* Upper limit of normality, *LLN* Lower limit of normality

After a screening period of approximately 6 weeks, which included a 4-week diet stabilization phase, patients were treated with 20 mg of vupanorsen administered subcutaneously every week for 26 weeks and then followed for 13 additional weeks (post-treatment follow-up period).

The study was conducted at the University of Michigan. Written informed consent was obtained from all patients. The protocol was approved by the Institutional Review Board of Michigan Medicine and complied with the Declaration of Helsinki. The study was registered at ClinicalTrials.gov (NCT03514420).

### Study endpoints

All efficacy endpoints evaluated the change from baseline at Week 27 (one week after the last dose of the vupanorsen). In addition, an intermediate evaluation of all endpoints was performed at Week 13.

The primary endpoint was the percent change in fasting triglyceride levels at Week 27. Secondary endpoints included percent change and/or absolute change in: (1) ANGPTL3, fasting lipids and lipoproteins, including very-low density lipoprotein cholesterol (VLDL)-C, total cholesterol (TC), non-HDL-C, LDL-C, apolipoprotein (apo) C-III, apoB, apoB48 and FFA; (2) area under the curve (AUC) in lipid and glycemic parameters including triglycerides, FFA, plasma glucose, serum insulin, and C-peptide in response to a mixed meal test (MMT); (3) HbA1c and insulin resistance assessments including homeostasis model assessment of insulin resistance (HOMA-IR), adipose tissue insulin resistance (ADIPO-IR), insulin sensitivity index (ISI); (4) levels of adiponectin and leptin; (5) hepatic fat fraction (HFF) as assessed by magnetic resonance imaging (MRI); and (6) body fat distribution evaluated by dual-energy X-ray absorptiometry (DEXA).

### Measurements

Pathogenic variants in known lipodystrophy genes were sought by Next Generation Sequencing (NGS) utilizing the Lipodystrophy Panel developed by the University of Chicago.

All laboratory samples (blood and urine) were measured with commercially available assays at MedPace Reference Laboratories (Cincinnati, OH) except the MMT data where analytes were measured by the Michigan Diabetes Research Center Chemistry Core Laboratory. For measurement of lipid parameters, fasted blood samples were collected. LDL-C levels were measured either by ultracentrifugation or precipitation. The same methodology was used for all time-points for each individual patient, except for patient 01 who had baseline measured by ultracentrifugation and all other timepoints by precipitation. VLDL-C levels were calculated either as TC- (LDL-C + HDL-C) or as triglycerides divided by 5, respectively. ANGPTL3 was measured by ELISA, apoB by Nephelometry, apoC-III by photometry and FFA by turbidimetry (additional details about assays for select parameters can be found in Supplemental file 1). Platelet count measurements were also performed at the clinical pathology laboratory (Michigan Medicine Clinical Pathology Laboratory) by an automated hematology analyzer, Sysmex XN-9100.

For the MMT, patients were advised to consume a standardized meal the evening before the test (750 kcal, 20 % of energy from protein, 30 % from fat, and 50 % from carbohydrate) and refrain from consuming alcohol for 72 h. A cannula was inserted into a forearm vein, and after overnight fasting (minimum 8 h), a venous blood sample was taken between 8:00 AM and 10:00 AM. Patients then consumed a liquid load of Optifast (Optifast; Novartis, Minneapolis, MN; 474 ml, 320 kcal, 50 % carbohydrate, 35 % protein, 15 % fat) within a 15-min period. Additional blood samples were taken at 10, 20, 30, 60, 90, 120, 150, 180, 240 and 300 min after meal consumption. For each time point, approximately 15 mL of blood was collected: 10 mL into a red top without anticoagulant and 5 mL into a green top tube with heparin. All samples were placed on ice and processed immediately in the onsite laboratory. All time points were used for generating the AUC.

Surrogate insulin resistance estimates (HOMA-IR [20] and Adipo-IR [21]) were calculated based on fasting insulin, glucose and FFA concentrations, and ISI [22] was calculated from the blood glucose and insulin levels in the fasting (0 min) and postprandial (120 min) conditions. Insulin resistance indices were measured by utilizing the following equations:

$$\text{H}\text{O}\text{M}\text{A}-\text{I}\text{R}= (\text{F}\text{a}\text{s}\text{t}\text{i}\text{n}\text{g} \text{g}\text{l}\text{u}\text{c}\text{o}\text{s}\text{e} \left(\text{m}\text{m}\text{o}\text{l}/\text{L}\right) \times \text{F}\text{a}\text{s}\text{t}\text{i}\text{n}\text{g} \text{i}\text{n}\text{s}\text{u}\text{l}\text{i}\text{n} (\text{m}\text{U}/\text{L}\left)\right)/22.5$$

$$\text{A}\text{D}\text{I}\text{P}\text{O}-\text{I}\text{R}=\text{F}\text{a}\text{s}\text{t}\text{i}\text{n}\text{g} \text{F}\text{F}\text{A} \left(\text{m}\text{m}\text{o}\text{l}/\text{L}\right)\times \text{F}\text{a}\text{s}\text{t}\text{i}\text{n}\text{g} \text{i}\text{n}\text{s}\text{u}\text{l}\text{i}\text{n} (\text{p}\text{m}\text{o}\text{l}/\text{L})$$

$$\text{I}\text{S}\text{I}=\text{l}\text{o}\text{g}(\text{m}\text{e}\text{a}\text{n} \text{i}\text{n}\text{s}\text{u}\text{l}\text{i}\text{n}({\upmu }\text{I}\text{U}/\text{L})/\text{M}\text{C}\text{R};\text{w}\text{h}\text{e}\text{r}\text{e} \text{l}\text{o}\text{g}(\text{m}\text{e}\text{a}\text{n} \text{i}\text{n}\text{s}\text{u}\text{l}\text{i}\text{n} ({\upmu }\text{I}\text{U}/\text{L}))=[(\text{l}\text{o}\text{g}(\text{f}\text{a}\text{s}\text{t}\text{i}\text{n}\text{g} \text{i}\text{n}\text{s}\text{u}\text{l}\text{i}\text{n} \left({\upmu }\text{I}\text{U}/\text{L})+\text{log}\left(2\text{h}-\text{p}\text{o}\text{s}\text{t} \text{p}\text{r}\text{a}\text{n}\text{d}\text{i}\text{a}\text{l} \text{i}\text{n}\text{s}\text{u}\text{l}\text{i}\text{n}{\upmu }\text{I}\text{U}/\text{L})/2\right.)\right]\text{a}\text{n}\text{d} \text{M}\text{C}\text{R} \left(\text{m}\text{e}\text{t}\text{a}\text{b}\text{o}\text{l}\text{i}\text{c} \text{c}\text{l}\text{e}\text{a}\text{r}\text{a}\text{n}\text{c}\text{e} \text{r}\text{a}\text{t}\text{e}\right)=\text{m}/\text{m}\text{e}\text{a}\text{n}\text{g}\text{l}\text{u}\text{c}\text{o}\text{s}\text{e}, \text{m}=[75,000+(\left(\text{f}\text{a}\text{s}\text{t}\text{i}\text{n}\text{g} \text{g}\text{l}\text{u}\text{c}\text{o}\text{s}\text{e} \left(\text{m}\text{g}/\text{L}\right)-2\text{h} \text{p}\text{o}\text{s}\text{t} \text{p}\text{r}\text{a}\text{n}\text{d}\text{i}\text{a}\text{l} \text{g}\text{l}\text{u}\text{c}\text{o}\text{s}\text{e} \left(\text{m}\text{g}/\text{L}\right)\times 0.19\times \text{w}\text{e}\text{i}\text{g}\text{h}\text{t} \left(\text{k}\text{g}\right)\right]/120\text{a}\text{n}\text{d} \text{m}\text{e}\text{a}\text{n} \text{g}\text{l}\text{u}\text{c}\text{o}\text{s}\text{e}=[\text{f}\text{a}\text{s}\text{t}\text{i}\text{n}\text{g} \text{g}\text{l}\text{u}\text{c}\text{o}\text{s}\text{e} \left(\text{m}\text{m}\text{o}\text{l}/\text{L}\right)+ \text{g}\text{l}\text{u}\text{c}\text{o}\text{s}\text{e} \text{a}\text{t} 2\text{h} \left(\text{m}\text{m}\text{o}\text{l}/\text{L}\right)]/2$$

Central imaging analysis of MRI data was performed at MedPace Imaging Clinical Laboratory. HFF MRI analysis was performed using scanners with 1.5T or a 3T field strength and proton density fat fraction (PDFF) acquisition software [23]. Whole-body composition, including fat and lean body masses, was evaluated by DEXA (GE Lunar Prodigy, model PA +41,744) [24].

Additionally, a complete medical history and a physical exam were performed monthly during clinical follow-up visits. During the clinical visit, lifestyle measures recommended for the treatment of this population were reinforced per the 2016 treatment guidelines for lipodystrophy [7], including nutrition counseling and physical activity, and patients were reminded to carefully follow the instructions.

### Safety monitoring

In addition to standard safety, monitoring and stopping rules for platelet count, liver and renal function were pre-specified in the protocol. The platelet count was monitored every 2 weeks and analyzed simultaneously by both central and local laboratories. Liver and renal function tests were monitored every 2 weeks during the first 3 months of the treatment period and monthly thereafter. Per protocol, the investigator could interrupt or permanently discontinue study treatment for any safety reason, including clinically meaningful safety signals in any clinical laboratory results.

### Statistical analysis

For this small, single-arm proof of concept study, descriptive statistics were used to summarize the observed and change from baseline data. The AUC of lipid and glycemic parameters, in response to MMT, was calculated from the curves obtained from the values plotted over time using the linear trapezoidal method.

## Results

### Patient population

A total of 8 patients with FPLD were screened, and 4 patients were enrolled and treated with vupanorsen for 26 weeks. The reasons for screen failure were: diagnosis of diabetes mellitus made less than 6 months from screening (2 patients), an estimated glomerular filtration rate lower than 60 mL/min/1.73m^2^, and liver function tests >2 times ULN. All enrolled patients completed the study.

Demographic and baseline characteristics for each patient are presented in Table 2. The mean age was 42.2 ± 3.7 years (range: 38-47 years), and 3 patients were female. Genetic causes of FPLD were identified in 2 patients, showing different *LMNA* gene mutations. In the other two patients, since there were no pathogenic variants in the known lipodystrophy genes, they were classified as FPLD Type 1. All 4 patients had family history of abnormal and similar fat distribution. Baseline laboratory values are presented in Tables 3 and 4. Baseline mean ± SD triglyceride level was 9.24 ± 4.9 mmol/L (817.8 ± 431.9 mg/dL), and, except for HDL-C, all other mean baseline lipids and lipoprotein parameters were elevated. By protocol design, patients also had elevated HbA1c (9.5 ± 1.3 %) and HFF (19.7 ± 9.6 %). Baseline leptin and adiponectin levels (shown in Table 2) were within the range expected from the underlying diagnosis of FPLD.

**Table 2:** Demographics and baseline characteristics of patients

Demographics/baseline characteristics	Patient 01	Patient 02	Patient 07	Patient 08
**Age (years)**	43	41	38	47
**Sex**	Female	Male	Female	Female
**Race**	White	White	White	White
**BMI (kg/m**^**2**^**)**	24.7	33.5	33.6	30.9
**FPLD diagnosis confirmed by genetics**	*LMNA* R482Q	No	No	*LMNA* R584H
**Family History of Abnormal and Similar Fat Distribution**	Yes	Yes	Yes	Yes
**Required a High Dose of Insulin**	No	Yes	No	Yes
**Acanthosis Nigricans**	Yes	Yes	No	Yes
**PCOS or PCOS-like symptoms**	Yes	NA	Yes	Yes
**History of Pancreatitis Associated with Hypertriglyceridemia**	No	Yes	No	Yes
**Non-alcoholic Fatty Liver Disease**	Yes	Yes	Yes	Yes
**Leptin (µg/L)**	6.8	28.2	19.7	16.9
**Adiponectin (mg/L)**	3.3	0.8	5.2	4.5

*BMI* Body mass index, *FPLD* Familial partial lipodystrophy, *PCOS* Polycystic ovary syndrome

**Table 3:** Change from baseline in fasting ANGPTL3, lipid and lipoprotein levels with treatment

Study parameters Mean (SD)	Baseline	Week 27	Change from baseline (%)
**ANGPTL3 (µg/L)**	115.4 (28.0)	53.9 (22.8)	-54.7 (9.8)
**Triglycerides (mmol/L)**	9.2 (4.9)	3.4 (3.0)	-59.9 (26.3)
**Apo C-III (g/L)**	0.3 (0.1)	0.1 (0.1)	-50.9 (27.4)
**VLDL-C (mmol/L)**	2.7 (1.0)	1.3 (0.8)	-53.5 (18.6)
**Non-HDL-C (mmol/L)**	5.8 (0.6)	4.6 (1.4)	-20.9 (17.7)
**Total cholesterol (mmol/L)**	6.4 (0.5)	5.3 (1.3)	-17.7 (14.8)
**LDL-C (mmol/L)**	2.9 (1.0)	3.3 (0.9)	19.2 (16.1)
**HDL-C (mmol/L)**	0.7 (0.1)	0.7 (0.2)	8.4 (26.0)
**Apo B (µmol/L)**	2.4 (0.5)	2.5 (0.6)	1.5 (24.0)
**Apo B48 (mg/L)**	51.1 (5.6)	15.1 (8.9)	-69.2 (19.8)
**Free fatty acids (mmol/L)**	1.1 (0.8)	0.5 (0.4)	-41.7 (40.6)

*ANGPTL3* Angiopoietin like 3, *Apo* Apolipoprotein, *VLDL-C* Very-low-density lipoprotein cholesterol, *HDL-C* High-density lipoprotein cholesterol, *LDL-C* Low-density lipoprotein cholesterol

**Table 4:** Changes in area under the curve from baseline during the mixed meal test in different metabolic parameters during the study

AUC	Baseline	Week 13	Week 27	Change from baseline to week 27 (%)
Triglycerides (mg/dL*min)	199120.0 (96813.0)	94270.0 (49145.2)	88022.5 (75031.1)	-60.1 (26.5)
Free Fatty Acids (mmol/L*min)	215.8 (55.2)	211.9 (75.5)	144.2 (65.4)	-32.1 (21.4)
**Glucose** (mg/dL*min)^a^	76882.5 (16771.4)	65265.0 (11720.3)	64558.7 (11431.1)	-14.0 (5.2)
**Insulin** (mIU/L*min)^a^	18790.1 (2499.7)	26972.7 (12877.3)	23440.0 (5329.6)	16.7 (19.1)
**C peptide** (ng/mL*min)^a^	1761.5 (498.9)	1580.5 (890.9)	1484.1 (698.6)	-11.6 (16.6)

Conversion factor for SI units: Triglyceride=0.0113 (mmol/L); glucose=0.0555 (mmol/L); insulin=6 (pmol/L); C peptide=0.33 (nmol/L)

Data are presented as mean (SD). *AUC* area under the curve, *SD* standard deviation

^a^One patient (008) with unstable and difficult to control diabetes requiring treatment with high doses of U500 insulin was excluded from analysis of FPG and parameters related to circulating insulin

### Change from baseline in fasting triglycerides, ANGPTL3, and other lipids and lipoproteins with treatment

**Fig. 1:**
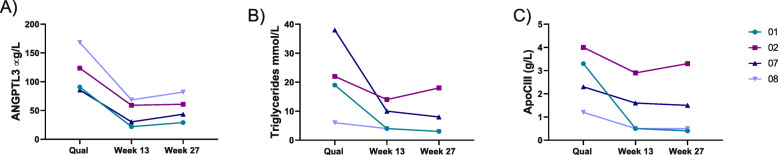
Effect of vupanorsen on ANGPTL3 (**A**), Triglyceride (**B**), and VLDL-C (**C**) levels in individual participants over the course of the study. Legend shows the patient number to link individual data from different figures

Treatment with vupanorsen resulted in lowering of serum ANGPTL3 by a mean of 54.7 % (Table 3). This result was accompanied by a mean reduction in levels of fasting triglycerides (by 59.9 %), VLDL-C (by 53.5 %), non-HDL-C (by 20.9 %), apoC-III (by 50.9 %), apoB48 (by 69.2 %), and FFA (by 41.7 %). Reductions in these parameters were already observed at Week 13 in all patients (Fig. 1). LDL-C levels showed a small non-significant increase during observation period, and no effect of vupanorsen was observed on apoB or HDL-C (Table 3).

### Effect of vupanorsen treatment on fasting plasma glucose, HbA1c, and insulin resistance indices

**Fig. 2:**
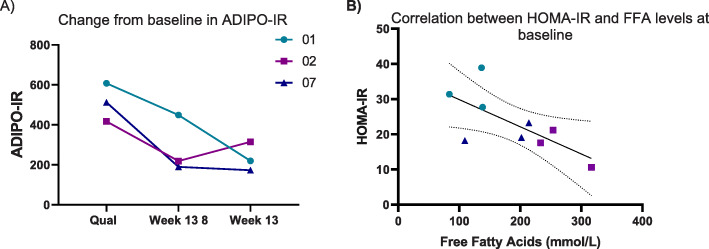
Change in insulin resistance indices during the study. **A** Adipo-IR (adipose tissue insulin resistance index) and **B** correlation between HOMA-IR (homeostatic model assessment- insulin resistance index) and free fatty acids. Legend shows the patient number to link individual data from different figures

Fasting plasma glucose (FPG) showed a decrease from baseline by 3.2 ± 0.7 mmol/L at Week 27 and there was no significant effect on HbA1c levels (Table 5). Treatment with vupanorsen reduced the adipose tissue insulin resistance as measured by ADIPO-IR by 55 % while HOMA-IR and ISI showed no change (Table 5; Fig. 2A). However, at baseline, the HOMA-IR values were inversely associated with serum FFA (Fig. 2B). One patient (08) had difficult to control diabetes, requiring treatment with very high doses of U500 insulin, and was excluded from analyses of FPG and other parameters involving circulating insulin and glucose levels.

**Table 5:** Change in fasting plasma glucose, HbA1c and insulin sensitivity indices from baseline with treatment

Parameters* Mean (SD)	Baseline	Week 13	Week 27	Absolute change from baseline to week 27
FPG (mmol/L)	13.1 (1.1)	11.7 (1.1)	9.8 (0.6)	3.2 (0.7)
HbA1c (%)	9.5 (1.3)	9.3 (1.1)	9.3 (1.1)	-0.2 (0.9)
HOMA-IR	27.1 (6.8)	28.6 (16.5)	22.3 (7.7)	-4.0 (0.7)
ADIPO-IR	470.3 (114.3)	385.8 (230.9)	216.3 (70.6)	-209.3 (120.4)
ISI	0.045 (0.007)	0.039 (0.004)	0.037 (0.001)	-0.0039 (0.002)

### Change in postprandial lipid and glucose metabolism as measured during the mixed meal test with vupanorsen

**Fig. 3:**
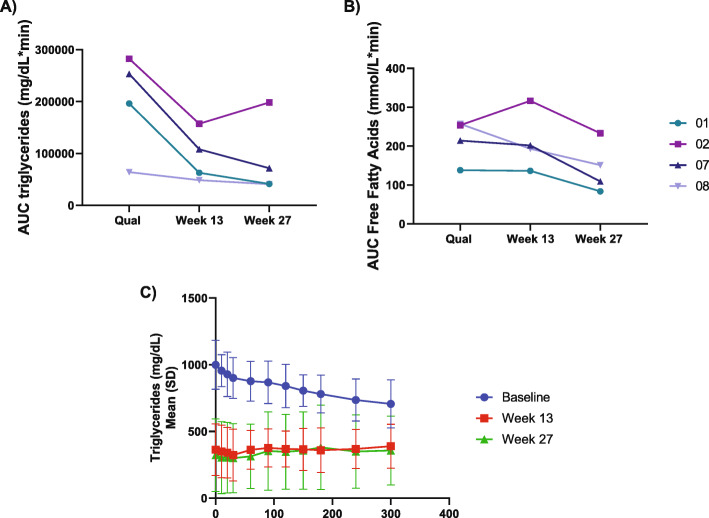
Postprandial response in the area under the curve (AUC) of triglyceride (**A**) and free fatty acid (FFA) (**B**) levels in the individual patients, and mean triglyceride levels from all participants (**C**) during the mixed meal test at key study time points. Legend shows the patient number to link individual data from different figures. Conversion factor for SI units for triglycerides=0.0113 (mmol/L)

At baseline, triglyceride levels did not rise postprandially; and were in fact slightly reduced in 3 out of 4 patients (Supplemental file 2). Following treatment with vupanorsen, the MMT at Week 27 showed decreases in AUC for postprandial triglycerides and FFA levels compared to baseline of 60 % and 32 %, respectively (Table 4). The individual values of AUC for triglyceride levels (Fig. 3A) showed a consistent effect of vupanorsen observed in all patients, with a progressive reduction in AUC with treatment over time. A similar pattern was observed for FFA in 3 out of 4 patients (Fig. 3B). Reductions in mean postprandial triglyceride levels before and after vupanorsen are shown in Fig. 3C. Small decreases in mean AUC for glucose and C-peptide were observed, while insulin AUC showed a trend toward an increase (Table 4, Supplemental file 2).

### Changes in HFF, body fat distribution and fat cell hormones

**Fig. 4:**
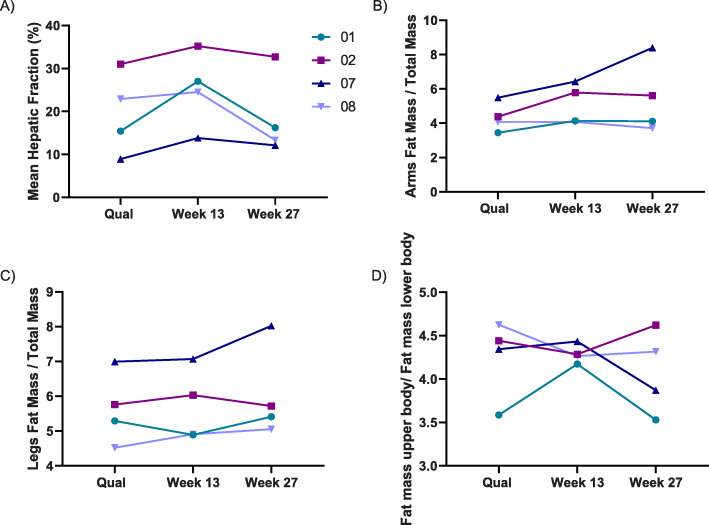
Change from baseline in hepatic fat fraction (**A**) and body fat distribution (**B**, **C** and **D**) during the study in individual patients. Legend shows the patient number to link individual data from different figures

The mean HFF displayed a specific pattern during vupanorsen treatment, with an increase from baseline of 19.7 ± 9.6 % (mean ± SD) to 25.1 ± 8.7 % (absolute increase from baseline by 5.4 ± 4.4 %) at Week 13, followed by a subsequent decrease to 18.5 ± 9.5 % (absolute decrease from baseline by -1.1 ±1.2 %) at Week 27. This trend was observed in all 4 treated patients (Fig. 4A).

There was no change at Week 27 in the amount of fat in the trunk or total body, however a trend towards an increase in the extremity fat (Fig. 4B and C) was observed. The ratio of fat mass in the arms to total body fat increased by 15.6 ± 18.8 % while the ratio of the fat mass in the legs to total body fat also showed a numeric increase of 6.2 ± 6.4 %. The ratio of the fat mass from limbs to the fat mass in the trunk showed an increase of 10.7 ± 10.6 % (Fig. 4D). Other body composition parameters remained unchanged throughout the treatment period, including body weight and BMI. Interestingly, while the study could not quantify objective data on the amount of neck fat, there were clinical changes in the consistency and shape of the hypertrophied neck depots in patients 01 and 08 with *LMNA* variants, with subjects reporting easier neck movement and less pressure. No effect was observed on leptin or adiponectin levels with treatment (Supplemental file 3).

### Safety and tolerability

The 4 patients reported a total of 90 adverse events (AEs) during the study, among them 70 AEs (77.8 %) were considered mild in severity, and 18 (20 %) were considered as related to the Study Drug, with the majority related to hepatic steatosis and liver enzyme increases (discussed below). Three patients experienced a total of 8 serious adverse events (SAEs), all assessed as not related to the Study Drug and recovered. These were: ventricular tachycardia and angina pectoris that occurred during, and following, elective dobutamine stress test in a patient with a history of chest pain, palpitations, and family history of premature coronary artery disease (CAD); acute pancreatitis in a patient with a history of pancreatitis: atrial flutter, atrial fibrillation with either troponin increase or pulmonary edema, respectively, and acute coronary syndrome in a patient with diffuse 3 vessel CAD not amenable to revascularization. No patient discontinued treatment due to AE, 3 patients had short interruptions of the Study Drug either due to SAEs (2 patients), or, in the case of the first patient enrolled, due to AE of hepatic steatosis with transient elevation of HFF that was an unexpected finding at the time it was observed. Treatment was resumed after an assessment by a hepatologist, and despite treatment continuation, HFF returned to baseline value.

Overall, transient increases in transaminases to >2 and <3 times ULN (or <2 times baseline value in a patient with elevated baseline) were observed in 3 patients early during the study, with peak values at Week 4-13, and returned to normal values thereafter despite continuation of treatment. These increases in transaminases seemed to accompany increases in HFF and were not associated with changes in bilirubin or INR levels.

One patient reported mild injection site reaction (dryness), and there were no reports of flu-like reactions. There was no effect of vupanorsen on platelet count (no platelet value below LLN), and no clinically significant changes in renal function tests.

## Discussion

In this proof-of-concept study in patients with genetic or clinical diagnosis of FPLD, treatment with the novel therapeutic vupanorsen targeting *ANGPTL3*, resulted in a robust reduction in fasting triglycerides associated with a reduction in ANGPTL3, VLDL-C, non-HDL-C, and apoC-III levels. These effects were like those reported for vupanorsen in patients with diabetes, hepatic steatosis and hypertriglyceridemia [25]. Reductions in apoB48, postprandial triglycerides, fasting glucose, fasting and postprandial FFA levels, and ADIPO-IR index were also observed. In addition, changes in HFF and DEXA parameters suggested potential dynamic changes in fat partitioning. These effects of vupanorsen were independent of genetic confirmation of diagnosis of FPLD [9], as they were observed in patients classified as FPLD2 with mutations in the LMNA gene, as well as in patients with FLPD1 with no causative single genetic variants identified in known lipodystrophy genes.

Hypertriglyceridemia is an important metabolic problem in patients with FPLD and should be a key clinical endpoint while searching for effective therapies. The precise mechanism(s) for the elevation of serum triglycerides in FPLD is still unknown, but it may be related to an increase in energy intake, ectopic lipid deposition particularly in the liver leading to enhanced VLDL secretion, or increased hepatic *de novo* lipogenesis [26, 27]. Hypertriglyceridemia may contribute to the high prevalence of cardiac disease among patients with FPLD, including early cardiac mortality [26, 28]. The reductions in fasting levels of triglycerides, TRLs and other atherogenic lipoproteins observed in this study suggests that vupanorsen may provide therapeutic benefit for patients with FPLD.

This study is one of the first to report triglyceride levels after MMT in patients with FPLD. It is important to note that more pronounced abnormalities were observed in patients with FPLD in the fasting state compared with the postprandial state. This observation highlights and supports the importance of hepatic *de novo* lipogenesis in metabolic dyslipidemia associated with lipodystrophy [29]. Treatment with vupanorsen was also associated with reduced AUC for postprandial triglyceride levels in response to MMT in all four patients. This may suggest more effective lipid uptake and oxidation by the tissues in response to the drug.

Treatment with vupanorsen also resulted in reduced levels in both, fasting and postprandial FFAs, consistent with previous reports on ANGPTL3 deficiency [16] and suggesting suppression of adipose tissue lipolysis. However, it has been debated why, or even if, FFAs are elevated in FPLD (and in other human lipodystrophy syndromes) due to the limited fat compartments and the expectation that lipolysis cannot increase in the fasting state [30]. It has also been questioned whether elevated FFAs are a measurement artefact due to *in vitro* breakdown of the triglycerides via the white blood cells. In this study, samples were kept on ice and processed rapidly to minimize potential deterioration. In addition, the authors have previously reported increased *in vivo* lipolysis even in patients with generalized lipodystrophy [27]. This raises the possibility that lipolysis may occur in alternative tissues via ectopic lipases to compensate for reduced lipolysis in the adipocyte compartment. However, more direct data are required to determine the source of increased lipolysis and FFA levels in individuals with FPLD.

Overall adiposity was stable after treatment, but careful analyses of the DEXA parameters and liver fat suggest that there may be dynamic changes in lipid partitioning between compartments such as liver and adipose tissue. In general, it is now accepted that the adipose tissue serves as a metabolic buffer protecting tissues such as the liver, pancreas and the heart from ectopic steatosis. The heterogeneous nature of adipose tissue and the many roles for the cells that compose this tissue in its various depots throughout the body have been reported [28, 31, 32]. It has been postulated that different subtypes of lipodystrophy may be associated with loss of different cell populations in the adipose tissue. Consequently, the hypertrophied depots in FPLD may arise from different and metabolically dysfunctional cell types. Also, the communication between the adipose tissue and liver is likely altered in FPLD. Therefore, it is possible that in FPLD, the response of the adipose tissue to ANGPTL-3 inhibition may be different from that in individuals with healthy fat depots. In this study, we observed a tendency for an increase in peripheral fat depots and a clinical change in the shape of the hypertrophied neck depots, especially in the two patients with LMNA variants, although this latter effect could not be quantified. The transient increase in HFF at Week 13, with return to baseline at Week 27 with vupanorsen treatment could be a consequence of changes in fat distribution. All of these changes taken together may suggest mobilization of lipid and a shift in partitioning versus normal adipose tissue; however, further studies with animal models or *in vitro* models such as 3D cell culture [33] are needed to better understand these observed changes. Further, the number of patients and the short treatment duration make it hard to reach definitive conclusions and these observations are reported here as potential hypotheses.

There was no evidence that treatment with vupanorsen affects whole-body glucose utilization, as no changes in HOMA-IR, ISI or HbA1c levels were observed, suggesting that the effect of the drug is not on a pathway that is downstream of Glut-4 translocation.

The observed reduction in ADIPO-IR, a measure of insulin resistance of the total adipose tissue compartment calculated from a single measurement of FFAs and insulin concentrations [34], likely reflects a decrease in circulating FFAs after vupanorsen treatment. The ADIPO-IR has been reported to correlate with hepatic fat content, a marker of ectopic fat deposition [35], and reduction in the index is usually coupled with improvements in non-alcoholic steatohepatitis histopathology in more common settings [36]. The observed reductions in fasting glucose plasma levels, despite no apparent change in HbA1c levels, may suggest an indirect effect of vupanorsen, secondary to changes in lipid partitioning and reduction of hepatic *de novo* lipogenesis. Since these are indirect effects and depend on certain changes in lipid dynamics to occur first, longer studies would be needed to provide support for these initial hypotheses generated by the current data.

The only drug that has been approved for treatment of lipodystrophy is metreleptin, a recombinant analogue of human leptin used as leptin replacement therapy to treat the metabolic complications of generalized lipodystrophy, but not that of FPLD [37, 38]. Previous studies have shown a decrease of approximately 20 % in triglycerides levels after 12 months of metreleptin in patients with partial lipodystrophy [4, 39]. Interestingly, metreleptin has also been shown to reduce elevated ANGPTL3 levels observed in generalized lipodystrophy. However, this effect was relatively small (~20 % reduction) and not correlated with changes in triglycerides or glycemia, hence probably not responsible for the overall metabolic benefits of metreleptin [40]. Although beyond the scope of this study, the robust reduction in triglyceride raises the question whether the two drugs can have additive effects in patients with FPLD which is a question for future studies. It is important to note that the patients with FPLD in the current study had baseline circulating leptin and adiponectin levels that were in the range previously reported for FPLD [6]. No change in leptin or adiponectin levels was observed upon treatment with vupanorsen, suggesting that the endocrine function of the adipocytes was not impacted by the drug.

The number of AEs observed in this study is consistent with the diverse pathologies and disease burden in patients with FPLD, and this reflects the common serious metabolic and organ specific manifestations of the disease. Treatment with vupanorsen was well tolerated and not associated with any changes in platelet count, consistent with the overall experience with GalNAc3 ASOs [41]. The open-label nature of the study and the lack of a control group limits the conclusions that can be reached on safety and a large placebo-controlled study of vupanorsen in patients with dyslipidemia is ongoing (NCT 04516291).

### Comparison with other studies and what the current work adds to existing knowledge

Treatment of FPLD has been very challenging due to the heterogenous pathophysiology of this disease, and this is reflected by the lack of approved therapies for FPLD. This is the first study to investigate a new therapeutic strategy for FPLD by inhibiting ANGPTL3 with vupanorsen. Overall, the observation of reductions in triglycerides, TRLs and other atherogenic lipoproteins levels, circulating FFAs along with the subsequent improvements in adipose tissue insulin resistance, and intriguing effects on fat redistribution in this proof-of-concept study highlight the need for further investigation of this treatment option in patients with FPLD.

### Study strength and limitations

Strengths of the study include (1) detailed baseline clinical characterization of enrolled patients in order to select a representative population of FPLD, and (2) extensive characterization of the effect of inhibition of ANGPTL3 with vupanorsen on a variety of metabolic parameters relevant to the complex pathophysiology and characterization of the disease. Limitations of this work include the open-label design, lack of a control group, and the small number of patients treated. In addition, only one dose was studied, and larger pharmacodynamic effects may have been observed with a dose leading to greater ANGPTL3 knockdown. However, these limitations are inherent to the nature of drug development at this early stage in a rare disease population.

## Conclusions

The results of this study suggest that targeting of ANGPTL3 with vupanorsen could address several key metabolic abnormalities in patients with FPLD. Further studies are necessary to evaluate the long-term benefit and safety of this treatment. Vupanorsen treatment in this patient population can serve as an interesting clinical model to probe modulation of lipid partitioning in humans and highlights the relative importance of lipid partitioning in regulating energy metabolism.

## Supplementary Information

**Additional file 1.**

**Additional file 2.**

**Additional file 3.**

## Abbreviations

ADIPO-IR: Adipose tissue insulin resistance
ANGPTL3: Angiopoietin-like protein 3
Apo: Apolipoprotein
AUC: Area under the curve
BMI: Body mass index
DEXA: Dual-energy X-ray absorptiometry
FFA: Free fatty acid
FPLD: Familial partial lipodystrophy
GalNAc3: N-acetyl galactosamine-3
Hb: Hemoglobin
HDL-C: High-density lipoprotein cholesterol
HOMA-IR: Homeostasis model assessment of insulin resistance
LDL-C: Low-density lipoprotein cholesterol
LLN: Lower limit of normal
MRI: Magnetic resonance imagining
Non-HDL-C: Non-high-density lipoprotein cholesterol
T2DM: Type 2 diabetes mellitus
TC: Total cholesterol
TRL: Triglyceride rich lipoprotein
ULN: Upper limit of normal
VLDL-C: Very-low-density lipoprotein cholesterol
