# Supplementary material for: Selective targeting of angiopoietin-like 3 (ANGPTL3) with vupanorsen for the treatment of patients with familial partial lipodystrophy (FPLD): results of a proof-of-concept study
Source: Lipids Health Dis. 2021 Dec 5;20:174. doi: 10.1186/s12944-021-01589-4 (PMC8647384; doi:10.1186/s12944-021-01589-4)
Supplement: Supplementary file 1 — Additional file 1. [file 12944_2021_1589_MOESM1_ESM.docx]

**Supplemental file 1:** Detailed laboratory methods for measurements of lipid parameters.

|  | **Method** | **Brand** | **Catalog number** | **Analytical Sensitivity** | **Assay Range** | **Inter-assay CV** | **Intraassay CV** |
| --- | --- | --- | --- | --- | --- | --- | --- |
| **ANGPTL3** | ELISA | R&D Systems Human | DANL30 | 8.2 pg/mL | 7.8-500.0 ng/mL | <12% | <10% |
| **Apo B** | Nephelometry | Siemens BNII nephelometer (Malvern, PA) | NA | caliber dependent | 12-400 mg/dL | <5% | <10% |
| **Apo C-III** | Turbidimetry | Beckman Coulter Analyzers (Sekisui) | 241871 | 0.2583 mg/dL | 0.94-22.0 mg/dL | <5% | <10% |
| **FFA** | Turbidimetry | Beckman Coulter Analyzers (HR Series NEFA-HR (2), Wako) | 999-34691  991-34891  995-34791  993-35191  276-76491 | 0.0101 mmol/L | 0.010- 2.000 mmol/L | <5% | <10% |

ANGPTL3: angiopoietin like 3; Apo: Apolipoprotein; FFA: Free fatty acid; CV: coefficient of variation; NA: not available
