# Supplementary figures and images for: Selective targeting of angiopoietin-like 3 (ANGPTL3) with vupanorsen for the treatment of patients with familial partial lipodystrophy (FPLD): results of a proof-of-concept study
Source: Lipids Health Dis. 2021 Dec 5;20:174. doi: 10.1186/s12944-021-01589-4 (PMC8647384; doi:10.1186/s12944-021-01589-4)

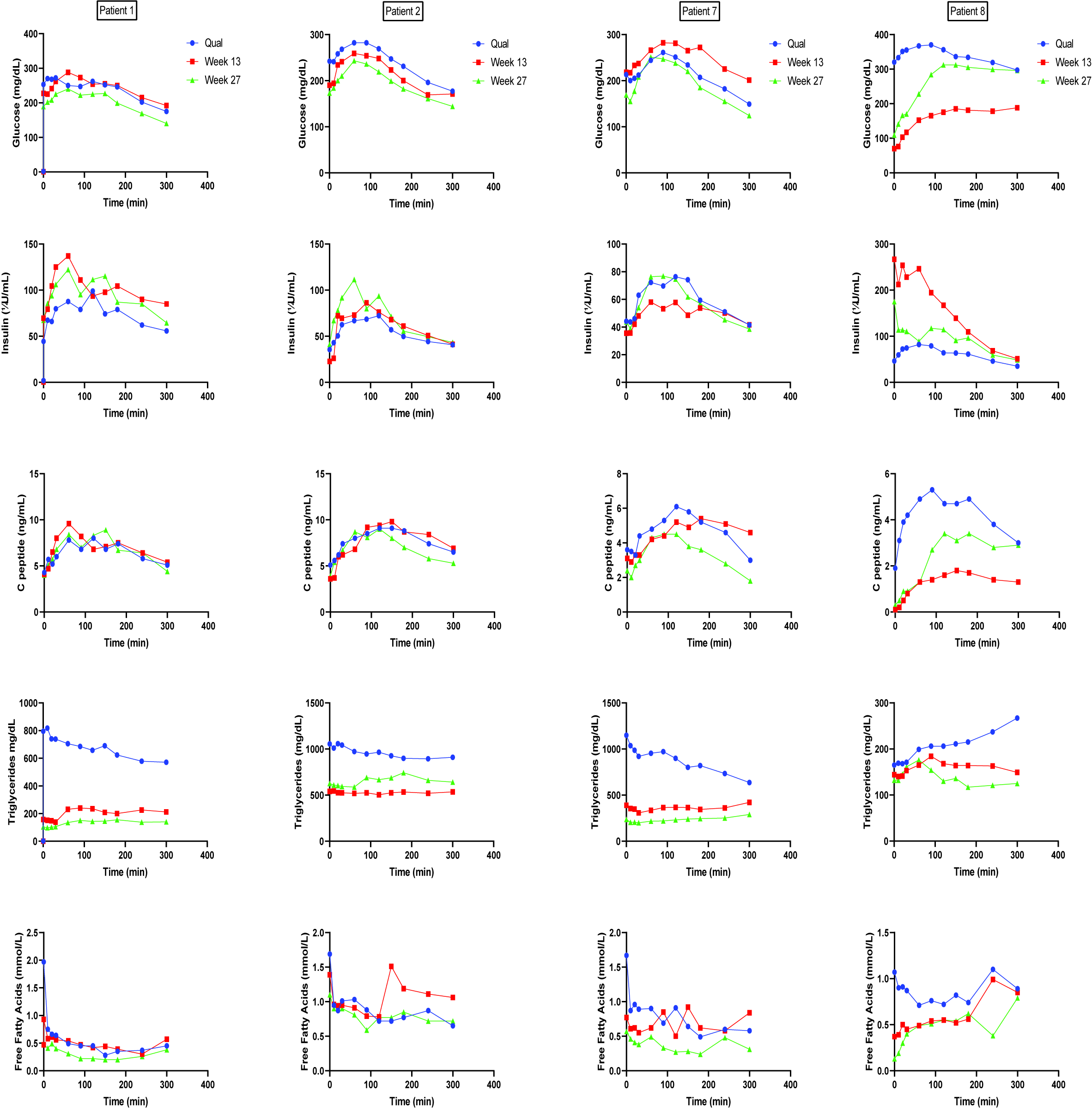

Supplement: Supplementary file 2 — Additional file 2. [file 12944_2021_1589_MOESM2_ESM.tif]

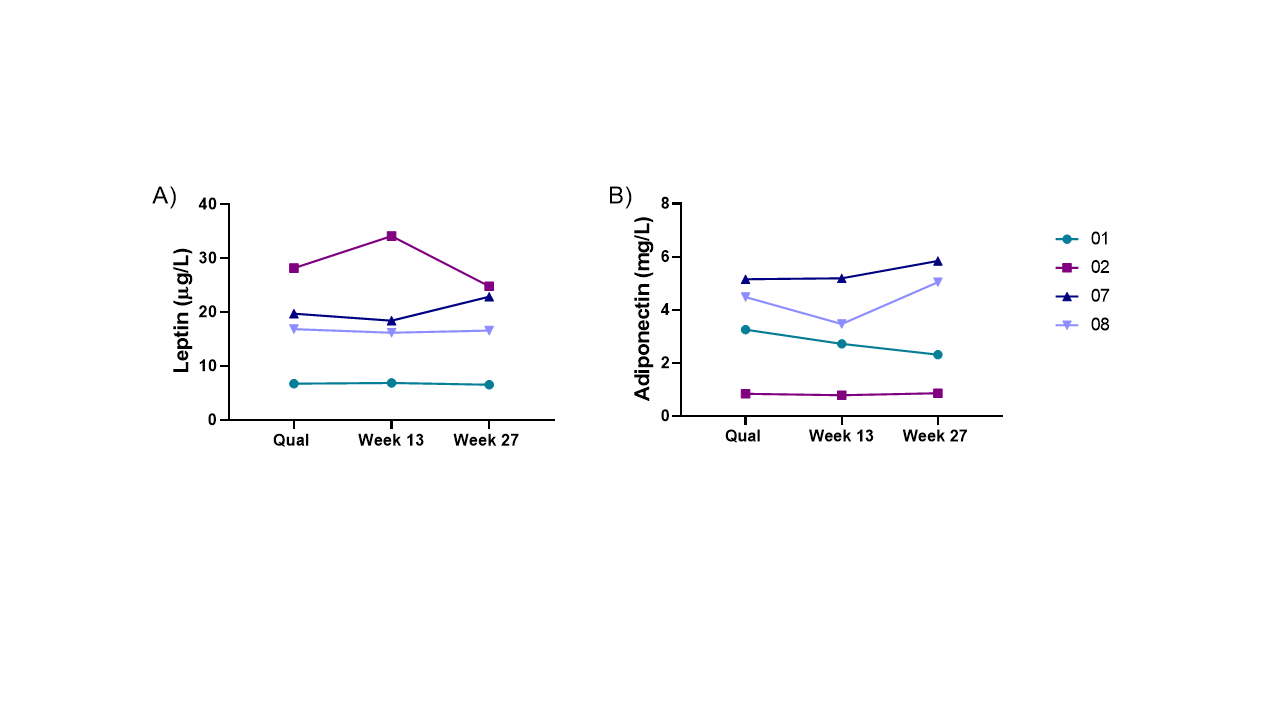

Supplement: Supplementary file 3 — Additional file 3. [file 12944_2021_1589_MOESM3_ESM.tif]
